# Role of Intravesical BCG as a Therapeutic Vaccine for Treatment of Bladder Carcinoma

**DOI:** 10.52547/ibj.3676

**Published:** 2022-11-08

**Authors:** Delaram Doroud, Hamidreza Hozouri

**Affiliations:** 1 Production and Research Complex, Pasteur Institute of Iran, Tehran, Iran;; 2 Department of Quality Management, Pasteur Institute of Iran, Tehran, Iran

**Keywords:** BCG, Bladder carcinoma, Bacterial products

## Abstract

Bacterial products have attracted much attention as potential antitumor agents, with the ability to provide direct tumoricidal effects, leading to the inhibition of tumor growth. Treatment of superficial bladder cancer with intravesical BCG has a more reduction potential than surgery in tumor recurrence rate. BCG, the gold standard for NMIBC, is manufactured from different strains and produced commercially with varied strengths. There are a few countries known as the manufacturer of this strategic biopharmaceutical product, and Iran as a member of the Eastern Mediterranean Region plays a vital role in supplying this vaccine. Studies have failed to uncover the exact mechanism of action of the intravesical BCG; however, evidence points toward an immunogenic mechanism that proficiently modifies a biologic response and provokes the immune cells in order to kill and suppress tumors. Among various underlying mechanisms, BCG bacillus attachment to fibronectin through its FAP is a pivotal mechanism for BCG tumoricidal activity.

## INTRODUCTION

Bacteria and bacterial products have been used as a potential therapy for cancers, with regard to tumor regression, in cases with a concomitant bacterial infection^[^^1^^]^. Nowadays, nonpathogenic (attenuated or genetically modified) bacterial species have received considerable interest as possible anticancer agents. This feature arises from their ability to exert direct tumoricidal actions or to transport tumoricidal chemicals, resulting in tumor growth inhibition. Evidence of cancer regression in patients with bacterial infection has led to the discovery of standard clinical therapies such as the use of BCG for treating the superficial bladder cancer^[^^2^^]^. 

In the early 1890s, an American surgeon named Dr. William Coley (reviewed in^[^^3^^]^) noticed that acute bacterial infection could cause cancer to regress. Based on this observation, he injected live bacteria into a patient with advanced cancer and then developed a safe and effective bacterial compound for cancer treatment^[^^3^^]^. A century later, mechanisms involved in tumor regression were discovered by nonspecific stimulation of the immune system and eventually gave rise to the discovery of the first standardized clinical therapies^[^^4^^]^.

The anticancer effect of tuberculosis was noted at the beginning of the 20^th^ century. In 1929, during a series of autopsies at Johns Hopkins Hospital in USA, Pearl reported a statistically significant reduction in cancer incidence in patients with tuberculosis (reviewed in^[^^5^^]^). In 1935, Holmgren^[^^6^^]^ first reported success in treating cancer following an experience of the intravenous injection of BCG to 28 patients suffering from stomach cancer. In the 1950s, the positive effect of BCG on the treatment of cancer in animals was confirmed^[^^7^^]^. However, using BCG to treat cancer remained silent until the 1960s. An investigation by Coe and Feldman^[^^8^^] ^in 1966 indicated a strong delayed-type hypersensitivity reaction to BCG in bladder, which increased the propensity to use BCG in cancer treatment. In 1969, Mathe *et al.*^[^^9^^] ^published a promising paper about the therapeutic effect of BCG on lymphoblastic leukemia. Although Mathe's findings have not been replicated, it has been shown that BCG is mostly effective when the tumor is highly immunogenic. Concurrently with advancement and efficacy of contemporary chemotherapy and radiation therapy, desire to utilize BCG as a drug decreased^[^^9^^]^. Incidentally, in the 1970s, the hypothesis of bacteria as an anticancer agent had strengthened again and in this regard, a clinical study was performed using the nonpathogenic strain M55, *Clostridium buttyricum*, to treat malignant brain cancers^[^^10^^]^. During that study, it was found that glioblastoma multiforme turns into tumor abscesses by injecting M55 strain spores, and in this case, the spores are easily removed from the brain. There were multiple reports in the same decade showing the positive effects of BCG on cancer treatment^[^^10^^,^^11^^]^. For the first time in 1976, Morales and associates^[^^11^^]^ reported the use of intravesical BCG in the treatment of superficial bladder cancer. They showed that the recurrence of this cancer in nine patients treated with intravesical BCG was 12 times lower than that of the control group. In 1980, Lamm^[^^12^^] ^confirmed Morales' observations and demonstrated that intravesical BCG significantly reduced recurrence and delayed the interval between relapses. In 1995, Akaza and colleagues^[^^13^^] ^evaluated the therapeutic effect of intravesical BCG on patients with carcinoma *in situ*. Years later, Dr. Taei (thesis, unpuplished data) used BCG manufactured by the Pasteur Institute of Iran (Production and Research Complex) in the Golestan Hospital in Ahvaz (Iran) to treat superficial bladder tumors. They compared the preventing effect of BCG with thiotepa, an anticancer chemotherapy drug classified as an alkylating agent that interferes with the growth of cancer cells in the recurrence of superficial bladder tumors. The other application of BCG is intralesional administration for treatment of metastatic melanoma^[^^14^^]^. Previous *in vitro* and *in vivo* studies have exhibited that BCG, in terms of adhesion to fibronectin, is capable of adhering to both fibronectin matrix in fibrin clots and bladder tumor cells. Fibronectin and BCG mediated by FAP irreversibly binds to fibronectin, indicating the important role of BCG in the attachment to the bladder wall^[^^15^^]^ .

**Anatomy and histology of bladder **

The bladder is a hollow, balloon-shaped organ located in the pelvis. This muscular organ is responsible for storing urine generated by the kidneys prior to excretion. The bladder lumen is an environment for urine storage and surrounded by many cell layers, comprising bladder wall layers. The innermost layer, which is in direct contact with urine, is called the mucosal layer or urothelium. Lamina propria or connective tissue forms the next layer. The third layer of this tissue is made up of smooth muscle^[^^16^^]^. 

**Bladder cancer**

**Fig. 1:**
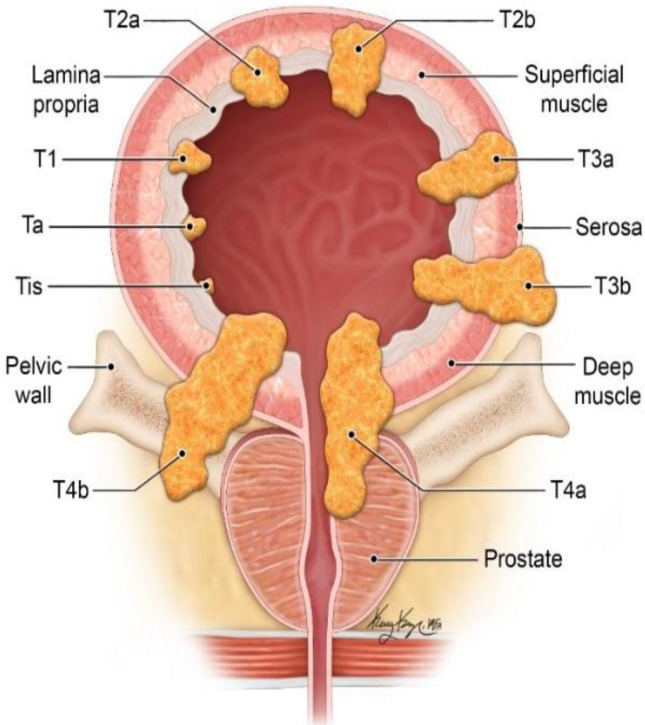
Outline of staging of bladder cancer from *in situ* carcinoma to invasive tumor^[^^20^^]^

Approximately 95% of bladder neoplasms arises from the mucosal epithelium. The rest mainly includes mesenchymal tumors of myoblastic, fibroblastic, or endothelial origin, which are similar to tumors in other parts of the body^[^^17^^]^. Most epithelial tumors are malignant and important for different reasons: they are relatively common, and although being histologically low in severity at the beginning, they could mostly be recurrent; as a result, their prognosis worsens, and monitoring and predicting their final situation become very difficult^[^^18^^]^. Almost 75% of bladder cancers diagnosed early are classified as superficial bladder cancer or NMIBC. The rest of the tumors develop as muscle-invasive bladder cancer^[^^19^^]^. Superficial bladder cancer is defined as a cancer confined to the first two layers of bladder tissue, the urothelium, and the lamina propria. A papillary tumor is a superficial tumor that extends into the bladder lumen. While CIS or TIS is found inside the cell layers and does not protrude, it is more likely to progress inward. Also, once the tumor travels to the smooth muscle and beyond, it is referred to as invasive bladder cancer^[^^20^^]^. Based on the results of diagnostic tests and biopsies, bladder tumors are divided into different stages based on their level of invasion (Fig. 1). As described in Table 1, superficial 

**Table 1:** Stages of superficial bladder cancer^[^^21^^]^

**Stages**	**Description**
T1	Invasive papillary tumor of the lower layer of the lamina proprietor (the tumor has invaded the connective tissue below the epithelium)
	
Ta	Tumor confined to the urothelium and extended into the lumen of the bladder (non-invasive papillary carcinoma)
	
CIS	*In situ* red lesions (limited to urothelium)

Bladder cancer includes three stages: T1, Ta, and CIS. Accurate determination of the stage and grade of bladder cancer is essential for correct diagnosis, treatment, and follow-up. The stage and grade of cancer is an indicator for the possibility of its recurrence and progression to an aggressive form. In 70% of instances with superficial bladder cancer, referred to as low-risk tumors, there is a chance of recurrence but no chance of tumor progression. While in the remaining 30%, which are high-risk tumors, there is an increased likelihood of the disease progressing to an invasive form. The division into low-risk and high-risk groups will be useful in predicting the course of the disease and treatment orientation, following up the disease, and determining the prognosis of the patient^[^^21^^]^.

**Types of bladder cancer**

Bladder cancer is categorized based on the type of cell involved as follows: transitional cell tumors, nontransitional cell tumors, and other tumors. Table 2 indicates the classification of tumors based on cellular characteristics, and the Table 3 shows the classification of bladder tumors and related subgroups^[^^22^^-^^24^^]^. 

***Transitional cell tumors***

These tumors are divided into benign (including exophytic and inverted papilloma) and malignant (including superficial bladder carcinomas)^[^^22^^]^.

*Benign-exophytic papilloma*

Papilloma is a benign and rare tumor. Under microscope, its cauliflower-like structure is covered by an epithelial layer. There is a possibility for the recurrence of this type of tumor after treatment, and in 13% of cases, it may change to a malignant tumor^[^^25^^]^.

*Benign-inverted papilloma*

This tumor with a cauliflower-like structure looks like a soft superficial gland and penetrates inversely into the bladder wall. Inverted papilloma accounts for less than 1% of all bladder urothelial neoplasms^[^^26^^]^.

*Malignant cell carcinoma*

The most common and important type of bladder tumor in Iran and the world is transitional cell carcinoma^[^^27^^]^. Table 4 depicts the presented statistics regarding the bladder cancer prevalence in Iran^[^^28^^]^. According to a study conducted in Iran from 2001 to 2007, the survival rate of the low-grade and high-grade urothelial cell carcinomas was 81% and 66%, respectively^[^^29^^]^. Malignant cell carcinoma as another kind of this tumor is divided into two general categories: invasive (to muscle) and noninvasive bladder carcinoma. 

Invasive bladder carcinoma

 Invasive bladder carcinoma is a malignant tumor invades the muscular layer of bladder wall (Fig. 1).

**Table 2:** Pathological grade of bladder cancer^[^^21^^]^

**Grade**	**Description**
I	Tumor are well differentiated, have papillary structure, healthy chromatin, and little evidence of nucleolysis or mitosis.
	
II	Tumors are relatively differentiated, usually have a papillary structure, chromatin is granular. There is stronger evidence of nucleolysis or mitosis.
	
*III*	Tumors are poorly differentiated, are less likely to have a papillary structure, coarse chromatin. There are many samples, nucleotides, or mitosis.

**Table 3:** Classification of bladder tumors^[^^23^^,^^24^^]^

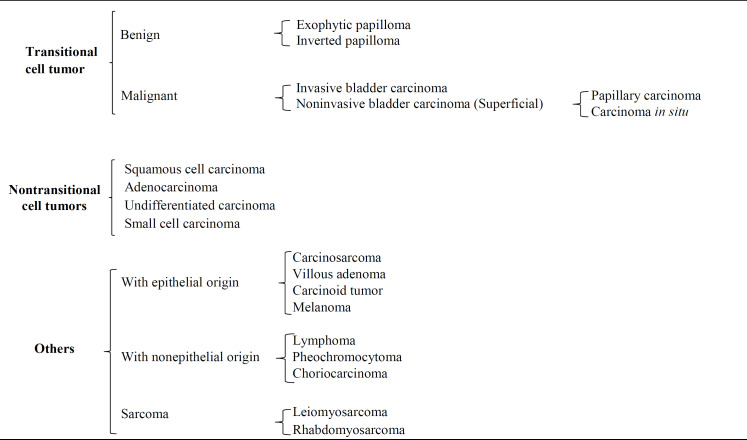

Noninvasive bladder carcinoma (superficial bladder carcinoma)

Noninvasive bladder carcinoma is a malignant tumor that has not penetrated into the muscular layer of the bladder wall, and its maximum penetration is up to the lamina propria. This type of carcinoma is important because it is the only bladder tumor that responds appropriately to intravesical medical treatment, especially intravesical BCG. In other words, intravesical therapy should be used only if the bladder cancer is superficial TCC. Superficial bladder carcinoma can be divided into two types: Papillary and carcinoma *in situ*. In the former carcinoma, the tumor often grows in the form of cauliflower inside the bladder cavity. However, the latter carcinoma is flat (without base) and superficial (without invading the bladder wall) with a high degree of malignancy. Under the microscope, throughout the thickness of the epithelial layer, changes in dysplasia and anaplasia can be observed as large cells with clear nuclei, without any damage to the lamina propria. This condition usually accounts for about 10% of TCC cases.

**Intravesical BCG** **efficacy and clinical studies **

For more than four decades, intravesical BCG was a highly efficient therapeutic approach for NMIBC^[^^30^^]^. The success of intravesical BCG treatment depends on its proper and timely use. Intravesical BCG can be utilized for various purposes, including (1) treatment of carcinoma *in situ* and papillary tumor remnants, (2) reduction of the bladder cancer recurrence, and (3) prevention of the disease progression. Intravesical BCG has been shown to be effective in the treatment of carcinoma *in situ* in 88% of patients with primary CIS (during the 44-month follow-up period) and 87% of patients with secondary CIS (during the 40-month follow-up period). In all of these patients, cytology and cystoscopy findings were negative. Intravesical BCG is less commonly used to treat the papillary tumors. Intravesical BCG has given a complete response to 66.4% of the patients with Ta or T1 stage tumors and a relative response to 8.20% of other patients^[^^31^^-^^33^^]^.

**Table 4:** Prevalence of bladder cancer in Iranian men and women^[^^23^^]^.

** Morphology**	**Transitional cell carcinoma**	**Squamous cell carcinoma**	**Adenocarcinoma**
**Gender**			
Male	2169 cases (95.8%)	23 cases (1%)	15 cases (0.7%)
Female	467 cases (96.8%)	5 cases (1%)	4 cases (0.8%)

**Intravesical BCG efficacy in bladder cancer recurrence**

Bladder cancer recurs in most patients with previous cancer disease. Therefore, by reducing or preventing the recurrence of superficial bladder cancer, both patients and health care systems can be helped. So far, many studies have been performed to compare the effect of different intravesical treatments on superficial bladder cancer^[^^34^^-^^39^^]^. However, it was difficult to compare these findings in terms of differences in the amounts of dosage and the variety of strains used in different brands. In Table 5, intravesical BCG brands are listed. Comparing the treatment of superficial bladder cancer with intravesical BCG with that of surgery as the only treatment (control group) displayed that intravesical BCG more reduces tumor recurrence rate[^40^^-^^43^^]^. Evidence has indicated that tumor recurrence is 75% in the control group and 31% in the treatment group. Furthermore, in a comparison between intravesical BCG and chemotherapy, it has been shown that chemotherapy lowers recurrence rates during the first five years of the disease, but beyond this time period, the recurrence rate is comparable to the control group who had only surgery. Overall, the recurrence of the disease remains low in patients treated with intravesical BCG^[^^40^^-^^43^^]^. Numerous studies have compared the therapeutic effect of intravesical BCG and chemotherapy drugs. In a study, 261 patients with superficial bladder cancer (during a 39-month follow-up period) were selected and randomly divided into two groups. The first group (group 1) was given intravesical BCG, and the second group (group 2) was prescribed mitomycin. The results showed that 49% of group 1 and only 34% of group 2 became tumor-free. Besides, in the first group, decrease in the number of recurrences of the disease and also increase in the time interval after which the disease begins to recur were significant. These results were confirmed by the Southwest Oncology Group. It was also found that in the first group, the disease recurred after 36 months and in the second group after 20 months^[^^40^^-^^43^^]^. Herr and colleagues^[^^41^^]^ showed the effectiveness of intravesical BCG together with transurethral resection method (group 1) in preventing the progression of tumor by comparing with the use of the transurethral resection method alone (group 2; control). They concluded that the diseases progression in the second group (95%) was more than the first group (53%). Invasion of muscle or metastasis in the first group was significantly delayed (*p* = 0.012), and cystectomy was required in 42% of the control group and 26% of the intravesical BCG-treated group. These results show a statistically significant difference (*p* = 0.017) between the two groups. Furthermore, the time interval between cystectomy requirements was 8 months in the control group and 24 months in the intravesical group. The mortality rate was reported to be 32% in the second group and 14% in the first group (*p* = 0.032)^[^^40^^]^. Lamm *et al.*^[^^42^^] ^compared doxorubicin with intravesical BCG and found that the tumor progressed in 37% of patients treated with doxorubicin and in 14% of patients treated with intravesical BCG (*p* = 0.017). Studies have shown the superiority of intravesical BCG in the treatment of superficial bladder cancer versus doxorubicin and thiotepa. Tumor progression was reported to be 1.5% in the intravesical BCG-treated group, 3.6% in the thiotepa-treated group, and 7.5% in the doxorubicin-treated group^[^^41^^-^^44^^]^. About 40% of patients who were candidate for receiving the intravesical BCG therapy found the experience of failure in treatment^[^^45^^]^. 

**Mechanism of action of intravesical BCG**

**Fig. 2:**
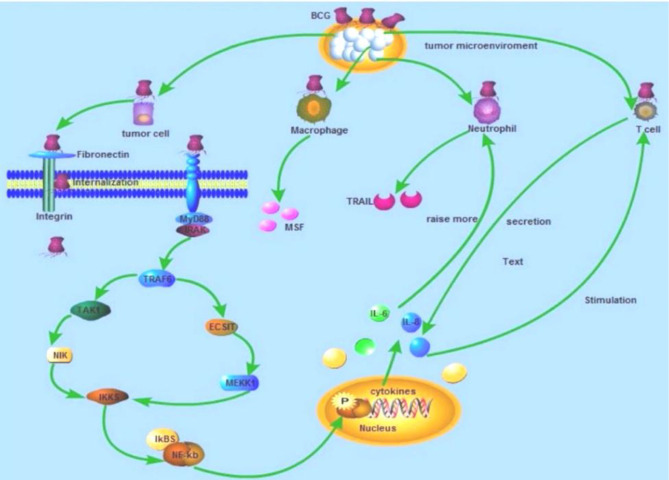
Mechanism of action of intravesical BCG in bladder cancer. Live BCG is attached to the urothelium via fibronectin and integrin and internalized by bladder cancer cells. As a result of simultaneous pathways, cytokines are secreted by neutrophils and macrophages, leading to the recruiting of immune cells to the site^[^^51^^]^.

Mechanism of action of intravesical BCG has not completely been known. However, it has been exhibited that BCG acts as a modifier of the biological response, guiding the immune system to kill and reduce tumors. Induction of apoptosis, necrosis, and oxidative stress are all potential stated assumptions^[^^46^^]^. In 1987, pioneering research by Ratliff and his colleagues^[^^47^^]^ signified the need for a complete immune system for BCG antitumor activities. In fact, they demonstrated that nude mice are unable to elicit an antitumor response in confronting BCG. Later in 1988, the same group displayed that BCG binds to bladder epithelial cells by an extracellular protein matrix called fibronectin, which covers the surface of the bladder and is the main ligand of BCG on epithelial cells^[^^47^^]^. BCG bacillus is attached to fibronectin via its FAP^[^^13^^]^. Fibronectin is assumed to bind to epithelium cells via α5β1 integrin^[^^48^^-^^50^^]^. Binding to fibronectin is essential for the antitumor activity of BCG^[^^33^^]^. After binding, BCG is endocytosed by epithelial cells (Fig. 2)^[^^50^^]^. BCG bacilli pass through the epithelial layer and activate pattern recognition receptor, which is exposed on the surface of bladder tumor cells. Activating the pathway of nuclear factor kappa-light-chain-enhancer of activated B cells promoted the transcription of cytokines. Antigen-presenting cells present processed BCG to CD4^+^ T cells and CD8^+^ T cells by binding to major histocompatibility complex class II in order to activate CD4^+ ^and CD8^+^. Moreover, CD4^+^ T cells, tumor cells, and innate immune cells secret copious cytokines following stimulation by BCG, which itself leads to the induction of apoptosis or cell death^[^^46^^,^^51^^,^^52^^]^.

**Table 5:** Characteristics of intravesical BCG brands^[^^34^^-^^39^^]^.

**Brand**	**Manufacturer**	**CFU/vial**	**Dosage form** **(mg/vial)**	**Diluent**	**Very common side effects (≥10%) **
OncoTICE® (Tice)	MSD (UK)	(2-8) × 10^8^	12.5	Saline	Cystitis, flu-like symptoms
					
OncoTICE® (Tice)	Merck (Canada)	(1-8) × 10^8^	50	Saline	Local irritation, flu-like symptoms, dysuria, frequent urination, hematuria, fever
					
TICE®BCG (Tice)	Organon (US)	(1-8) × 10^8^	50	Saline	Flu-like symptoms, dysuria, frequent urination, urgent urination, hematuria, fever, pain (not specified)
					
SII-ONCO-BCG (Russian)	Serum Institute of India	(1-19.2) × 10^8^	40	Saline	frequent urination, urgent urination, dysuria, cystitis, granulomatous, malaise, fever, flu-like syndrome
					
UROVAC (Danish 1331)	GreenSignal Bio Pharma (India)	(1-8) × 10^8^	40	Not reported	Not reported
					
Immunobladder (Tokyo 172)	Japan BCG Labaratory	Not reported	80	saline	Urodynia, frequent urination, hematuria, pyuria, dysuria, urgent urination, fever, proteinuria, leukopenia
					
BCG-Medac (RIVM derived from seed 1173-P2)	Medac GmbH (Germany)	(2-30) × 10^8^	80	Saline	Nausea, cystitis, granulomatous, frequent urination, prostatitis, fever, flu-like symptoms
					
PastoCys (Pasteur 1173P2)	Pasteur Institute of Iran	(5-30) × 10^8^	120	Saline	Not reported
					
Immucyst^*^ (Connaught)	Sanofi Pasteur (Canada)	(1.8-19.2) × 10^8^	81	Saline	Dysuria, residual urine, pyuria, cystitis, urinary tract infection, anemia, anorexia, nausea/vomiting, dysuria, frequent urination, hematuria, urgent urination, malaise, fever, chills, proteinuria
					
Theracys^*^ (Connaught)	Sanofi Pasteur (Canada)	(1.8-19.2) × 10^8^	81	Saline	Dysuria, nausea/vomiting, frequent urination, anorexia, malaise, renal toxicity (NOS), hematuria, genital pain, fever (>38 °C), chills, cystitis, anemia, urinary tract infection, urgent urination, flu-like syndrome

^*^Currently Sanofi Pasteur has stopped the production of these mentioned products

**PD** **-L1 and BCG therapy failure**

**Fig. 3:**
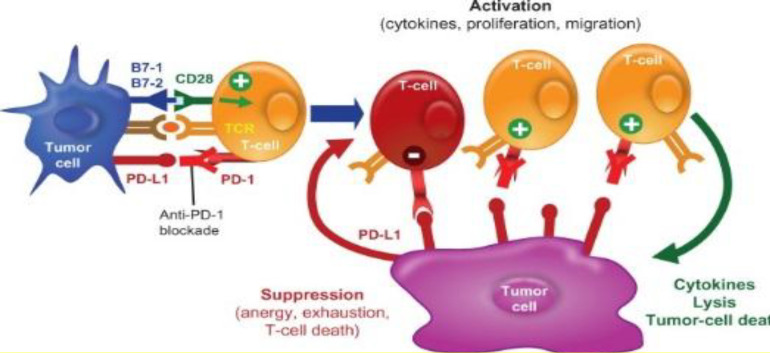
PD-L1 destructive role in effective BCG therapy response and T-cell benefits of anti-PD-L1^[^^58^^]^.

PD-L1 is express or induced in myeloid, lymphoid, normal epithelial, and tumor cells. The checkpoint acts as an essential mediator for suppressing immune cells in the tumor microenvironment. Indeed, the attachment of PD-L1 to PD-1 (receptor on T cells) enables tumor cells to deceive T cells into discounting the immune response (Fig. 3)^[^^53^^,^^54^^]^. Some new investigations have revealed that the unresponsive group of patients with CIS and NMIBC is due to the elevated PD-L1 expression on their tumor cells^[^^54^^,^^55^^]^. *In vivo* and *in vitro* studies have confirmed that the excessive expression of PDL-1 is a burden on efficient response to BCG therapy, which leads to treatment failure. Moreover, combination of BCG and anti-PD-L1 significantly increases the activity of CD8^+^ cells^[^^46^^,^^56^^,^^57^^]^. 

## DISCUSSION

Bladder cancer is the most common cancer of the urogenital system in Iran (ranked the second after prostate cancer) as compared to many parts of the world. Statistical studies have suggested that 70-80% of the initially diagnosed bladder cancers are superficial. In 1976, Morales *et al.*^[^^11^^]^ reported the use of intravesical BCG for treatment of superficial bladder cancer. This therapy is still used as the most successful treatment for superficial bladder cancer so that it was efficacious in 60-70% of cases; however, in some cases side effects are observed due to the consumption of high doses of the vaccine. Urologists have described which type of BCG is the most effective available product for treating the aggressive superficial bladder cancer using preclinical and immunologic studies. The **Pasteur Institute of Iran **is a regional leader in the field of developing this product, and with extensive experience and advanced platform, this institute offers the most effective strategy for development of BCG product in cancer treatment.

This vaccine remains the gold standard treatment for patients with intermediate- and high-risk NMIBC. After more than 40 years of use, the exact mechanism of action of BCG vaccine remains unknown, and further studies would be helpful to augment its efficacy and cost effectiveness. However, the future of BCG for preventing recurrences and progression in NMIBC looks still bright as no other effective therapy for these patients appears on the horizon, owing to the lack or absence of gold standard intravesical treatment. Different commercial brands manufactured by a few countries have different efficacy and adverse effects in cancer patients in accordance with different strains used with variable strengths. 

As the tremendous importance of the therapeutic effect of intravesical BCG as the first-line treatment for superficial bladder cancer and the compelling results of intralesional BCG administration in metastatic melanoma treatment, as well as its unique mechanism for the immune system stimulation, using bacteria, particularly BCG, as a potential treatment for cancer can be taken into account in future comprehensive studies.

## DECLARATIONS

### Ethical statement

Not applicable. 

### Data availability

The raw data supporting the conclusions of this article are available from the authors upon reasonable request.

### Author contributions

D.D: Prepared the first draft; H.H: searched data and revised the finalized version of manuscript.

### Conflict of interest

None declared.

### Funding/support

Not applicable. 
